# Screening for autoimmune thyroid disorders after spontaneous abortion is cost-saving and it improves the subsequent pregnancy rate

**DOI:** 10.1186/1471-2393-13-217

**Published:** 2013-11-22

**Authors:** Jana Bartáková, Eliška Potluková, Vladimír Rogalewicz, Tomáš Fait, Dita Schöndorfová, Zdeněk Telička, Jan Krátký, Jan Jiskra

**Affiliations:** 1 Third Department of Medicine, General University Hospital and First Faculty of Medicine, Charles University in Prague, Prague, Czech Republic; 2 CzechHTA, Faculty of Biomedical Engineering, Czech Technical University in Prague, Kladno, Czech Republic; 3 Clinic of Obstetrics and Gynecology, General University Hospital and First Faculty of Medicine, Charles University in Prague, Prague, Czech Republic

## Abstract

**Background:**

Hypothyroidism and/or autoimmune thyroid disorders (AITD) may contribute to spontaneous abortions (SpA). Cost-effectiveness analyses of thyroid screening in women after SpA are lacking. Our aim was to evaluate the cost-effectiveness of screening for AITD and/or hypothyroidism and their treatment in women after SpA with regard to their reproductive health.

**Methods:**

We performed a cross-sectional non-randomized study with follow-up in 2008–2011 in the settings of Departments of Endocrinology and Obstetrics/Gynecology of a university hospital. We enrolled 258 women after SpA before the 12th gestational week and followed them for a median of 3 years. At enrollment, serum concentrations of thyroid stimulatory hormone (TSH), antibodies to thyroid peroxidase (TPOAb) and free thyroxine (FT4) were measured and thyroid ultrasound performed. Women with overt hypothyroidism were treated with levothyroxine (n = 45; 61.6%) and women with subclinical hypothyroidism or euthyroid AITD were treated (n = 28; 38.4%) or left untreated (n = 38; 14.7%). Euthyroid women without signs of AITD served as controls (n = 147; 57.0%).

**Results:**

Of the 38 untreated women with AITD and/or subclinical hypothyroidism, 8 (21.1%) reported secondary infertility as compared to 16/147 (10.9%) controls and 3/73 (4.1%) treated women (p = 0.021). Treatment was associated with an increased rate of successfully completed subsequent pregnancies (increment of 6 newborns/100 women) and a savings of €19,539/100 women. Total costs per successfully completed pregnancy were €1,189 in controls, €1,564 in the treated, and €2,488 in the untreated women.

**Conclusions:**

Screening for thyroid disorders in women after SpA and treatment with levothyroxine is cost-saving and it improves the subsequent pregnancy rate.

## Background

About 10–15% of pregnant women are positive for autoantibodies to thyroid peroxidase (TPOAb) [1-3] and up to 5%, depending on the cut-off used, have elevated thyroid stimulating hormone (TSH) [4,5]. Untreated maternal thyroid disease during pregnancy may have a negative impact on the course of pregnancy and the development of cognitive function of the offspring [6,7]. Women with elevated TPOAb could have increased rates of infertility [8], miscarriage [9-11] and perinatal death [12]. Additionally, diagnosis and treatment of women with unrecognized hypothyroidism and euthyroid autoimmune thyroid disease (AITD) seem to be effective in preventing repeated spontaneous abortions (SpA) [13,14]. However, laboratory screening for thyroid disorders immediately after SpA is not recommended, except in cases of a subsequent pregnancies [15]; and in women after recurrent SpA in clinically suspected cases [16]. Although studies have been published on the cost-effectiveness of the thyroid screening in pregnant women [17-19], no study has assessed the clinical importance and cost-effectiveness of screening for thyroid disorders in women after SpA until now.

The aims of our study were: A) to evaluate the course and outcome of a subsequent pregnancy in women with AITD and/or hypothyroidism after SpA with regard to treatment with levothyroxine (LT4); and B) using a cost-effectiveness analysis to assess the suitability of laboratory screening for AITD and/or hypothyroidism in women after SpA from the perspective of women’s reproductive health (the time to conceive, natural vs. medically assisted conception, physiological delivery in term, premature delivery, caesarean section - SC, SpA, secondary infertility).

## Methods

### Patients and controls

The study was performed in the settings of Departments of Endocrinology and Obstetrics/Gynecology of the General University Hospital and the First Medical Faculty of the Charles University in Prague. It was designed as a cross-sectional non-randomized study during the post-abortion follow-up.

In years 2008–2011, serum concentrations of TSH, TPOAb and free thyroxine (FT4) were investigated and thyroid ultrasound (TUS) performed in 297 consecutively chosen women after SpA before the 12^th^ gestational weeks. Serum FT4 was measured only if TSH and/or TPOAb were abnormal. Median time of examination was 4 weeks after SpA. The investigated group consisted of all women who had SpA at the time of this project. The study was approved by the local Ethical Committee and all women signed an informed consent form (Ethics Committee of the General University Hospital, Prague).

**Figure 1:**
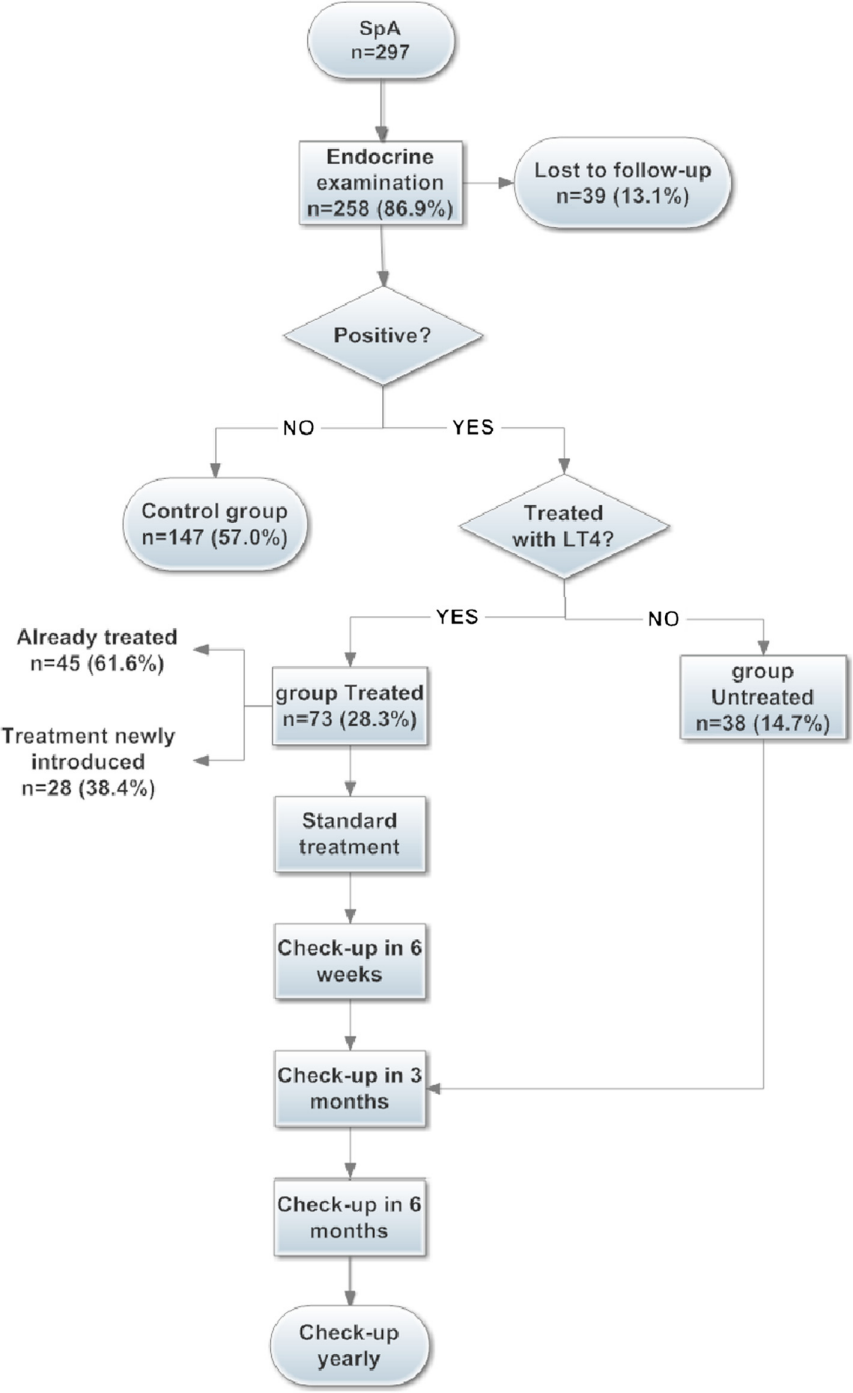
**Flowchart of standardized endocrine follow-up of women after spontaneous abortion.** Retrospective view. SpA, spontaneous abortion; LT4, levothyroxine; AITD, autoimmune thyroiditis.

We created a standardized scheme of endocrine care of women after SpA for the purposes of the study (Figure 1). Based on the findings in serum levels of TSH, TPOAb, FT4 and TUS, we decided whether to treat them with LT4. As positive in screening, women already treated with LT4 for thyroid diseases and women with newly diagnosed positive laboratory finding and autoimmune pattern on TUS were regarded. Overt hypothyroidism was defined as serum TSH >4.0 mIU/l with decrease in FT4 (<9.8 pmol/l); subclinical hypothyroidism as TSH >4.0 mIU/l and normal serum FT4 (9.8-23.1 pmol/l) and euthyroid AITD was defined as normal TSH (0.4-4.0 mIU/l) with markedly positive TPOAb (>254.4 kIU/l) or borderline positive TPOAb (60–254.4 kIU/l) and autoimmune pattern in TUS. Cut-off for markedly positive TPOAb was determined as 90^th^ percentile of serum concentration within group of 297 unselected included women. Borderline positivity was defined as TPOAb concentration between upper limit of manufacturer (60 kIU/l) and the 90^th^ percentile (254.4 kIU/l). LT4 treatment was obligatorily started in overt hypothyroidism and subclinical hypothyroidism with positive TPOAb. In euthyroid AITD and subclinically hypothyroid women with negative TPOAb, LT4 treatment was started on individual basis. In these cases the treatment was started if at least one of the following criteria were met: TSH >10 mIU/l; autoimmune pattern on TUS; and symptoms of hypothyroidism; or if the patient wished to be treated. Every endocrine check-up included a visit in the office of the endocrinologist (except in case of a check-up within six weeks, which was done by a telephone consultation) and a laboratory test of TSH. At the time of follow-up, 73 women were treated with LT4 for hypothyroidism or euthyroid AITD (group *“Treated”*, median age 33 years). From them, 45 women have been already treated and in 28 the treatment was newly introduced after SpA (8 for hypothyroidism and 20 for euthyroid AITD). There were no women after thyroidectomy or without signs of thyroid autoimmunity at TUS and negative TPOAb in group *Treated*. Thirty-eight women had euthyroid AITD and/or subclinical hypothyroidism without treatment (group *“Untreated”*, median age 32 years). Finally, 147 women were negative in thyroid laboratory screening and TUS (*“Controls”*, median age 33 years). Range of the standardized initial dosage of LT4 was 50–75 ug/day (median 51.34 ug/day). At the time of follow-up in the *Treated* group the laboratory tests were done. The target TSH (0.5-2.5 mIU/l) was achieved (median 1.36 mIU/l, range 0.52–2.37 mIU/l). There were no cases of overtreatment. For simplicity, further adjustment of the LT4 dosage to achieve the target TSH was not considered in the economic analysis.

At follow-up, we interviewed the women by telephone and we filled a questionnaire on the women’s history and reproductive health (19 questions including the time to conceive, natural vs. medically assisted conception, physiological delivery in term, premature delivery, SC, SpA, secondary infertility). As a physiological delivery at term, delivery in the 38^th^–42^nd^ gestational weeks was elected. Premature delivery was defined as delivery before the end of 37^th^ gestational week. For the purposes of our study, secondary infertility was defined as a lacking success in attempted conception during the time between the last SpA and data collection and analysis.

Although the Czech Republic belongs to the iodine sufficient countries [20], we advised all women after the reference SpA to use iodine supplementation of 100–150 ug/day before and during next pregnancy.

### Laboratory methods

Serum concentrations of TSH, TPOAb and FT4 were determined by chemiluminiscence method (ADVIA® Centaur™, Bayer, Germany). The reference intervals were determined by the manufacturer (TSH: 0.40-4.00 mIU/l, TPOAb: <60 kIU/l and FT4: 9.80-23.10 pmol/l). Using the manufacturer’s cut-off level for TPOAb, we found 36.4% of the women to be positive for TPOAb. Overall, 55.0% of the women had a positive laboratory finding (pathological TSH and/or TPOAb). Therefore, we decided to use a different upper cut-off limit for TPOAb: 254.4 kIU/l, as determined by Springer as the 90^th^ percentile of TPOAb values in a group of 5520 pregnant women [3].

### Thyroid ultrasound

The study participants underwent TUS examination within 9 weeks after SpA. We used the ultrasound device EnVisor (Philips) with an 8–12 MHz linear probe (model PLF-805ST), allowing maximum examination depth of 40 mm at a frame rate of 19 Hz. In order to eliminate the inter-individual variability, TUS examinations were performed by only one experienced physician. Ultrasound evaluation was made on the basis of hypoechogenicity, irregular echo pattern and the presence of nodules. In case of thyroid texture, we used our previously published semi-quantitative evaluation model [21]. Autoimmune pattern in TUS was determined as hypoechogenicity and inhomogeneity of the thyroid parenchyma.

### Statistical analysis

Statistical software Sigmastat (Jandel Scientific, San Rafael, CA, USA) was used for data analysis. The Chi-square test, Fischer test and ANOVA on ranks (Dunn’s method) were used to compare the proportions, means and medians between the groups. In order to assess the influence of the variables on the p-value in the Chi-square test in the contingency table, we used the Chi-square test of independence [22]. All reported p-values are two-side and p < 0.05 was considered as statistically significant. Throughout the text, data are expressed as mean (± standard deviation) or as median (range).

### Economic analysis

We used the cost-effectiveness analysis (CEA) for economic evaluation. CEA compares the costs and health effects of an intervention in order to assess the extent to which it can be regarded as providing value for money. The costs were estimated from the payer’s (health insurance) perspective. We determined the time horizon as the maximum time of the intended follow-up after SpA (four years prospective). Based on estimated published fiscal outlook of the Czech Republic’s Ministry of Finance [23], we assumed the discount rates to be 3% using 2012 unit costs. For the next years, we calculated the present value of a sum of a spent money by using discount rates and the time horizon [24]. We based the calculation of the discounting costs of end of pregnancy (physiological delivery in term; premature delivery; caesarean section; SpA) on the median time between the reference SpA and the end of the next pregnancy (Table 1). There were no differences in this median time between the groups. We discounted the costs of secondary infertility in the same way. The considered costs were defined as the medical costs directly related to treatment of an individual woman. The costs included medical visits (Depts. of Endocrinology and Obstetrics and Gynecology), laboratory tests and other examinations (e.g. ultrasonography, cardiotocography), administered drugs and days of hospitalization. We calculated the costs of medical check-ups, laboratory tests and examinations based on data extracted from the legislation administered by the Czech Ministry of Health (Decree No. 411/2011; Decree No. 439/2008; Decree No. 472/2009; Decree No. 425/2011) and we also used the payment algorithm (Decree No. 439/2008) of the same Ministry. In case of hospital care, we also included costs of hospitalization valid in the General University Hospital in Prague. We estimated the length of hospitalization as the average time linked to individual modes of pregnancy ending in our hospital. In order to calculate the prices of drugs administered, we used the database of The State Institute of Drug Control in the Czech Republic [http://www.sukl.cz/modules/medication/search.php?lang=1] and extracted the stated unit prices of drugs. All unit costs at 2012 prices are stated in Additional file 1.

**Table 1:** Total costs on the pregnancy outcomes

		**Physiological delivery**	**Premature delivery**	**SC**	**SpA**	**Secondary infertility**
Discount factor		1.000	1.000	1.000	1.000	0.971
Number of women	Treated	24	4	10	23	3
	Untreated	10	2	4	9	8
	Controls	52	8	24	20	16
Total unit costs (€)	Treated	389	402	493	287	1,516
	Untreated	346	359	450	244	1,473
	Controls	282	295	386	180	1,409
Total Costs (€)	Treated	9,334	1,608	4,929	6,607	4,547
	Untreated	3,805	718	1,799	2,442	11,782
	Controls	14,386	2,362	9,265	3,428	22,543

Discount factors were chosen on the relationship of the average time in the group of women after SpA (time from the reference SpA to the termination of subsequent pregnancy). There was no different in average time between the subgroups *Treated, Untreated* and *Controls*. The costs included the costs of screening for thyroid disease, regular endocrine controls, LT4 treatment and costs of pregnancy outcomes. Costs are calculated as a weighted average. Discount rate 3%, time horizon 4 years prospective, payer’s perspective. SpA, spontaneous abortion; SC, section cesarean.

We excluded women from the economic evaluation who did not try to get pregnant again after the SpA, as well as the currently pregnant women (we could not anticipate the pregnancy outcome). We considered a successfully ended pregnancy as a physiological delivery of a live newborn at term. We did not include costs due the newborn in the economic analysis.

### Health economic models

For estimation of the final costs and effects, we created a patient-based health economic model in which we tested the costs and effects of treatment and screening. The standardized endocrine follow-up of women after SpA is shown in Figure 1. Costs associated with pregnancy outcome (Additional file 1) are based on a model of a standardized process in the General University Hospital in Prague according to the actual outcome of the next pregnancies in groups analyzed (Table 1).

We calculated the assessment of costs related to treatment of an infertile woman with the use of the costs of the standard methods of infertility treatment used in our hospital. The In Vitro Fertilization (IVF) and the method of an Artificial Insemination by Husband (AIH) were calculated in the ratio of use 5:1. We calculated the average costs of ovulation stimulators per cycle of IVF (clomiphene or tamoxiphen) and average costs of follicle stimulating hormone per cycle in case of AIH.

In the analysis, we calculated the cost of four bed-days in the Dept. of Obstetrics and Gynecology in case of a physiological delivery; of five bed-days in case of preterm delivery and caesarean section (SC) and of one added bed-day in an Intensive Care Unit in case of SpA and SC.

All costs were converted to EUR with an approximate exchange rate in 2012 (1 CZK = 0.047 EUR).

## Results

From the 297 women included, 39 were lost to follow-up (one *Treated*, six *Untreated* and thirty-two *Controls*). At the time of follow-up (median 38 months, range: 8 – 47 months), 258 provided data concerning their subsequent reproductive health.

Of the 258 women analyzed, 111 (43.0%) were positive for thyroid disorders and 147 (57.0%) were negative. Of the 111 positive women, 45 (40.5%) had already been treated for hypothyroidism and/or AITD before inclusion in the study and 66 (59.5%) were newly diagnosed. Of the 66 newly diagnosed positive women, 15 (13.5%) had hypothyroidism (TSH >4.0 mIU/l) and/or markedly positive TPOAb, 26 (31.5%) had markedly positive TPOAb and normal TSH and 25 (22.5%) had borderline positive TPOAb and autoimmune pattern on TUS. Mean age at the current SpA was 32.52 ± 4.42 years and mean gestational age at the time of SpA was 9.15 ± 2.53 weeks. In 94 (36.4%) women, this was the first pregnancy; 118 (45.7%) had previous history of delivery of a live offspring and 91 (35.3%) had previous history of one or more SpA (70.3% one miscarriage, 22.0% two miscarriages, 7.7% three or more miscarriages). After the current SpA, the median of time to next conception was 7 months (range: 1 – 43 month) and the median of duration of secondary infertility was 26 months (range: 12 – 47). Baseline characteristics of women after SpA are shown in Table 2.

**Table 2:** Basal characteristics of the study participants at the time of spontaneous abortion

	**Treated with levothyroxine**		**Untreated**	**Controls**	**p-value**
	**Before SpA**	**Newly after SpA**			
**n**	45	28	38	147	
**Age (years)**	33	34	32	33	0.314
**BMI**	22.29	21.94	23.42	21.34	0.698
**Family history of thyroid disease**	19 (42%)	10 (36%)	14 (37%)	37 (25%)	0.118
**Autoimmune pattern in TUS**	10 (22%)	23 (82%)	30 (73%)	0 (0%)	<0.001
**FT4 (pmol/l)**	14.75 (11.70-22.20)	14.20 (11.30-20.60)	13.80 (11.00-17.50)	14.40 (11.10-20.20)	0.278
**TSH (mIU/l)**	1.84 (0.51-7.32)	2.26 (0.89-8.88)	1.91 (0.30-4.52)	1.54 (0.58-3.94)	0.002
**TPOAb (kIU/l)**	68.00 (15.00-4480.00)	118.00 (33.00-2820.00)	42.50 (22.00-1805.00)	41.00 (0.00-163.00)	<0.001
**Subclinical hypothyroidism**	-	8 (28%)	13 (34%)	-	0.827
**Euthyroid AITD**	-	20 (71%)	25 (66%)	-	0.827

The values are expressed as median (range). SpA: spontaneous abortion; TUS: thyroid ultrasound; FT4, serum concentration of free thyroxin; TSH, serum concentration of thyroid stimulating hormone; TPOAb, serum concentration of antibodies to thyroid peroxidase; AITD, autoimmune thyroid disorders. ANOVA on ranks (except women with family history of thyroid disease and autoimmune pattern in TUS: Chi-square test).

### Relationship of thyroid diseases and reproductive health

Thirty-one women who did not wish to conceive again and twenty women who were pregnant at the time of data collection were excluded from analysis.

The rates of secondary infertility among all positive women together (*Treated* and *Untreated*) and *Controls* were similar [11/111 (9.9%) vs. 16/147 (10.9%), p = 0.926]. Consistently, there were no significant differences of ability to conceive with methods of assisted reproduction between positive women and *Controls*.

*Treated* women had significantly lower rate of secondary infertility as compared to *Controls* and *Untreated* women [3/73 (4.1%) vs. 16/147 (10.9%) vs. 8/38 (21.1%), p = 0.021].

We did not find any significant effect of LT4 treatment on the frequency of subsequent SpA, SC and premature deliveries. Numbers of women in each group with respect to the subsequent pregnancy outcome are shown in Table 1.

### Costs

From the perspective of health insurance, unit costs of screening for thyroid disease in women after SpA (thyroid laboratory and TUS examination) were €27.05. The discounted unit costs from prospective of four years were calculated in the *Treated* group to be €150.94, in the *Untreated* group €107.88 and in the *Control* group €44.09 EUR. The costs included the costs of screening for thyroid disease, regular endocrine controls and LT4 treatment. Pregnancy outcomes and appropriate discount factors used depending on the median of the years in the group of women after SpA are shown in Table 1.

### Costs-effectiveness analysis

**Figure 2:**
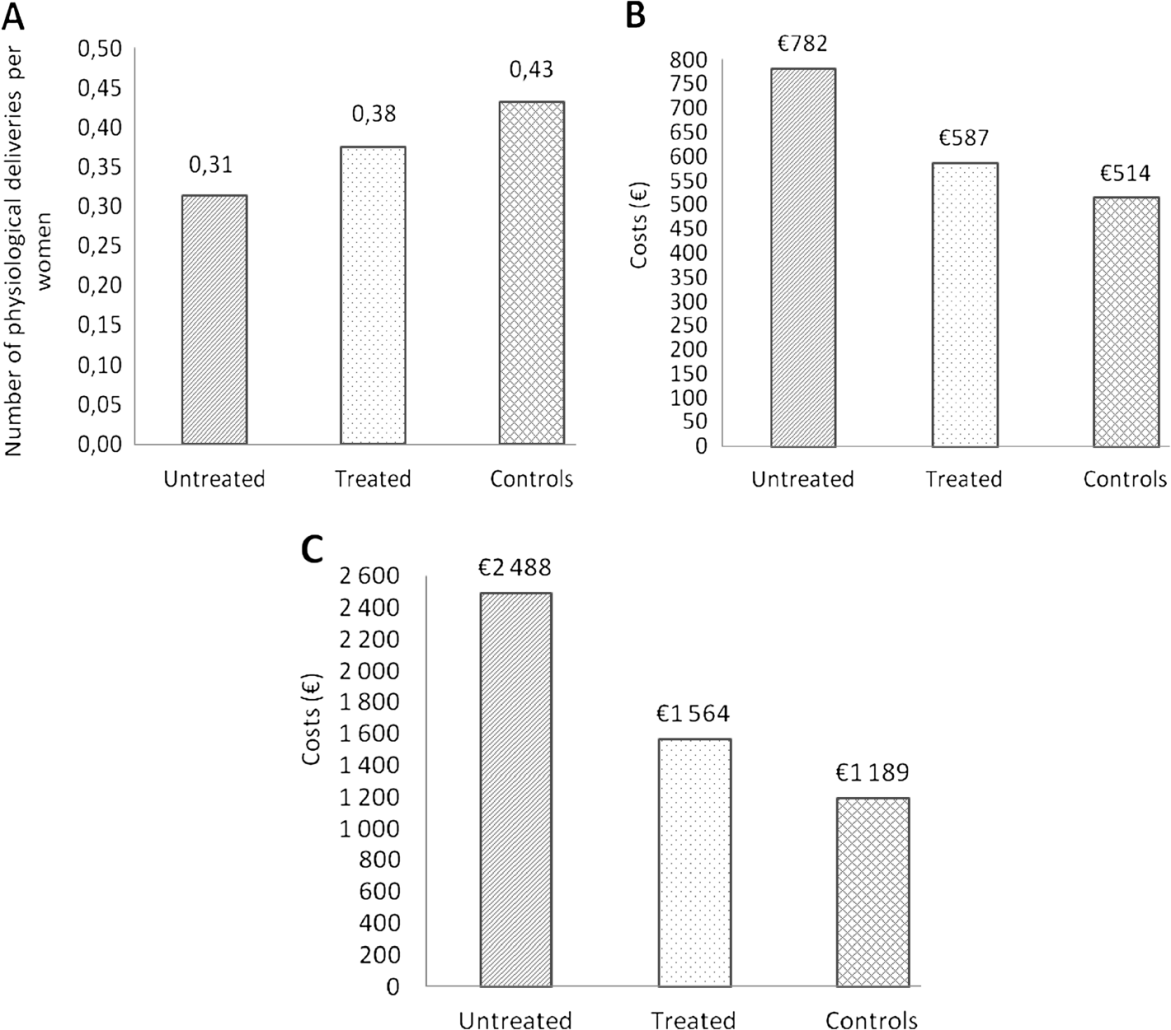
**Costs-effectiveness analysis. A**, Number of successfully completed pregnancies per women; **B**, total costs per women; **C**, costs per one successfully completed pregnancy.

Treatment of AITD and/or hypothyroidism was associated with an increased rate of successfully completed next pregnancies (increment of 6 newborns/100 women) and to savings of €19,539/100 women. Total costs per successfully completed pregnancy were €1,189 in *Controls*, €1,564 in *Treated*, and €2,488 in *Untreated* (Figure 2).

The total cumulative costs of healthcare associated with reproduction during four years in women with AITD and/or hypothyroidism were significantly lower if they were treated with LT4 (Table 1).

In the group of women after SpA, four patients (one *Treated* and tree *Controls*) had premature delivery with SC. In order to avoid double counting of costs for these patients, we subtracted the costs of thyroid screening, a possible LT4 treatment and follow-up endocrine controls and cost of premature delivery from the cumulative total costs of overall healthcare. Moreover, we added cost of methods of assisted reproduction in women who achieved further pregnancy with these techniques (eight *Treated*, five *Untreated* and seven *Controls*). We used an appropriate discount factor (0.971) depending on the median time to conceive again after SpA (2 years).

## Discussion

The recent years have witnessed a fierce debate on the topic of screening for thyroid diseases in pregnancy, including several cost-effectiveness analyses [17-19]. SpA belongs among the principal negative outcomes of untreated thyroid disorders in pregnancy. However, studies on thyroid screening in women after SpA in relation to the subsequent pregnancy are lacking. Our study presents the first cost-effectiveness analysis of thyroid screening in women after SpA.

Although women with pathological values in thyroid screening after SpA did not have a significantly higher rate of secondary infertility then women screened negatively, in the subgroup analysis targeted at LT4 treatment in women with AITD and/or hypothyroidism we achieved striking results: treated women had significantly lower rates of secondary infertility (21.1% vs. 4.1%) and increased rates of physiological deliveries (increment of 6 newborns/100 women) as compared to women untreated. Moreover, treatment with LT4 led to savings of €19,539/100 women from the time horizon of four years.

Our results are in line with findings of the recent randomized study of Kim et al. [25] who demonstrated that embryo implantation rate and live birth rate is significantly higher in women with subclinical hypothyroidism who were treated with LT4. Moreover, Zhung et al. evaluated 90 anti-thyroid antibody positive women and 676 anti-thyroid antibody negative infertile women undergoing IVF. They present evidence that the presence of anti-thyroid antibodies decreases fertilization rate, implantation rate and pregnancy rate [26]. On the contrary to the study of Negro et al. [13], we did not find significant beneficial influence of LT4 treatment on the preterm delivery and recurrent SpA, but our study was not designed to study these effects and it was not randomized. Similarly, in contrast to study of Negro et al. [27] who analyzed women undergoing IVF, we observed increased rates of physiological deliveries in women treated with LT4; however, our study wasn’t targeted at women undergoing IVF.

Although a number of studies have shown a link between thyroid disorders and an increased risk of recurrent SpA [28-30] and secondary infertility [29,31-33], we found only a non-significant trend to higher rates of secondary infertility or recurrent SpA among women with laboratory findings of AITD and/or hypothyroidism in comparison to women with normal laboratory results. This probably might be due to the fact that we analyzed women treated with LT4 already before SpA together with newly diagnosed positive women. When we analyzed previously just untreated positive women, we found significantly lower rate of secondary infertility in *Treated* group as compared to *Controls* and *Untreated* group. Moreover, rate of infertility was even lower in *Treated* group as compared to *Controls*. We suppose that this could be not only due to effect of levothyroxine treatment but, moreover, due to more accurate iodine supplementation in the *Treated* group in comparison with *Untreated* and *Controls*. Treated women were under the supervision of an endocrinologist and this fact could lead to more compliant using not only of levothyroxine, but also of iodine supplementation. As reported in literature, iodine supplementation before and during pregnancy can lead to increased fertility rate [34,35].

The main topic of our study was the cost-effectiveness analysis of screening for thyroid diseases in women after SpA. Based on time horizon of four years, we calculated the costs of screening, endocrine follow-up and treatment; and the costs of achieving the next conception leading to the successful delivery of a live newborn. The costs of single screening (TSH, TPOAb and TUS) was €27.05 per woman in our study; however, in routine practice the screening would probably be performed only by measuring TSH and TPOAb, thus decreasing the costs of a single screening to €18.36 per woman. The costs associated with long-term use of LT4 were only €8.98 per women per year. The costs of endocrine follow-up in positively screened women were €35.09 per woman for the first year and €13.21 per women per every additional year. Taken together, the costs associated with screening and treatment of AITD and/or hypothyroidism are very low as compared to treatment of infertility, where the highest costs were incurred by the methods of assisted reproduction. For example, one cycle of in vitro fertilization cost €1,586 per woman. Thus, it is evident that any decrease of infertility rates among women with thyroid disorders (achieved with LT4 treatment) may lead to truly significant financial savings. Moreover, our data show that LT4 treatment of AITD and/or hypothyroidism leads to an increase in successfully completed pregnancies (6 children/100 women; compared with untreated women) and to saving of €19,539/100 women from the time horizon of four years. It is thus apparent that LT4 treatment of women with AITD and/or hypothyroidism has a positive impact on their subsequent pregnancy rate; it is inexpensive and cost-saving.

Our study has several important strengths. Our health economic model used real data from patients, unlike the other cost-effectiveness studies [17-19] which use economic models based on transfer of results of other studies or expert estimates. It takes into account not only individual characteristics of patients, such a laboratory and TUS findings and the number of women undergoing assisted reproductive technology, but also specific characteristics which vary in different geographic area. Moreover, our calculation was based on the real costs accounted for in our hospital and stated by the Decrees of the Czech Ministry of Health. The cost-effectiveness of screening and treatment of thyroid disorders might be underestimated in our study, as we have not included some other potential benefits (e.g. improvement of the quality of life of treated women). Furthermore, even though the previously published studies demonstrated that the costs associated with premature birth are enormous [36], our calculated costs are low and approaching the costs incurred by physiological delivery. This is due to the fact that we evaluated costs related only to the treatment of the woman, but not to the neonatology care of the premature newborn, the increased morbidity of prematurely born children and also their further problems in learning and behavior [37].

Our study has several limitations. The main are the non-randomized design and a small number of women included (n = 258). Nevertheless, group consisted of all women who had miscarriage before the 12th gestational weeks at the time of this project. A further limitation is that the group of women treated before entering the study and the group of women with treatment introduced after SpA were analyzed together, similarly as untreated and treated positive women. The reason was to achieve an adequate number of women for statistical analysis. For all that, we deal with real costs associated with successful completion of the next pregnancy within the next four years after SpA. In addition, to make a clear economical model, we used simplification in case of LT4 treatment, where the further adjustment of LT4 dosage was not considered. However the costs of LT4 were minor (as was discussed above) and hence we anticipate that the omitting of future adjustment of LT4 dosage does not have a significant impact on the results of the study. We believe that our results provide a solid basis for future studies and considerations regarding the screening for thyroid disorders in women after SpA.

## Conclusion

In conclusion, our data indicate that screening for thyroid disorders in women after SpA and a consequent treatment with LT4 improve the subsequent pregnancy rate of women affected. Treatment with LT4 may reduce the necessity of methods of assisted reproduction in achieving the next pregnancy. Therefore, inclusion of systematic screening of TSH and TPOAb in the standard of care of all women after SpA might not only return the invested financial means of health insurance, but even lead to savings in long-term indicators.

## Competing interests

The authors declare that they have no competing interests.

## Authors’ contributions

JB was responsible for the data-collection , the analysis and interpretation of data. JB wrote, together with EP and JJ, the manuscript. JJ and VR supervised data analysis, statistical analysis and helped in interpreting results. VR helped draft and implemented the economic model and JJ with EP the health model. ZT handled the database and extracted data on patient’s history. JB, JJ, JK, TF a DS developed the original concept for the study and the study design. All authors have read and approved the final manuscript.

## Pre-publication history

The pre-publication history for this paper can be accessed here:

http://www.biomedcentral.com/1471-2393/13/217/prepub

## Supplementary Material

Additional file 1: **Unit costs of healthcare services.** The costs for medical check-ups, laboratory tests and other examinations were calculated by using payment algorithm (Decree No. 439/2008) and by using data extracted from the legislation administered by the Czech Ministry of Health (Decree No. 411/2011; Decree No. 439/2008). The costs of drugs administered were calculated by using the database of The State Institute of Drug Control in the Czech Republic (http://www.sukl.cz/modules/medication/search.php?lang=1). The costs of bed-days are the current costs applied in the in General University Hospital in Prague. SpA, spontaneous abortion; SC, cesarean section; CAR, Center of Assisted Reproduction; AIH, artificial insemination by husband; IVF, in vitro fertilization; CRP, C-reactive protein. Click here for file
