# Supplementary material for: Screening for autoimmune thyroid disorders after spontaneous abortion is cost-saving and it improves the subsequent pregnancy rate
Source: BMC Pregnancy Childbirth. 2013 Nov 22;13:217. doi: 10.1186/1471-2393-13-217 (PMC4222272; doi:10.1186/1471-2393-13-217)
Supplement: Additional file 1: — Unit costs of healthcare services. The costs for medical check-ups, laboratory tests and other examinations were calculated by using payment algorithm (Decree No. 439/2008) and by using data extracted from the legislation administered by the Czech Ministry of Health (Decree No. 411/2011; Decree No. 439/2008). The costs of drugs administered were calculated by using the database of The State Institute of Drug Control in the Czech Republic (http://www.sukl.cz/modules/medication/search.php?lang=1). The costs of bed-days are the current costs applied in the in General University Hospital in Prague. SpA, spontaneous abortion; SC, cesarean section; CAR, Center of Assisted Reproduction; AIH, artificial insemination by husband; IVF, in vitro fertilization; CRP, C-reactive protein. [file 1471-2393-13-217-S1.doc]

Additional file 1. Unit costs of healthcare services.

| **Category** | | **Unit** | **Cost (€)** | **Applicable in:** |
| --- | --- | --- | --- | --- |
| **Endocrine examination, treatment and follow-up** | | | | |
| Medical visit | Entrance endocrine examination | | 17.04 | All study participants |
|  | Telephone consultation with a doctor | | 3.24 | Treated participants |
|  | Follow-up endocrine examination | | 7.78 | Treated and Untreated participants |
| Laboratory test | Thyroid stimulating hormone (TSH) | | 5.43 | All study participants |
|  | Autoantibodies against thyroid peroxidase (TPOAb) | | 12.93 | All study participants |
| Other examination | Ultrasound examination of one organ | | 8.69 | All study participants |
| Drugs | levothyroxine (50 ug/day) | | 0.02 | Treated participants |
| **Examinations, follow-up and treatment in pregnancy** | | | | |
| Medical check-up | | Follow-up examination by a gynecologist | 5.78 | CAR (AIH, IVF) |
|  | | Detailed examination by a gynecologist | 10.95 | Physiological delivery, preterm delivery, SC, SpA |
|  |  | Detailed examination by an internist | 13.61 | SpA |
|  | | Vaginal delivery | 140.89 | Physiological delivery, preterm delivery |
|  |  | Caesarean section | 147.08 | SC |
| Laboratory test | | Follicle-stimulating hormone (FSH) | 5.02 | CAR (AIH, IVF) |
|  |  | Luteinizing hormone (LH) | 5.02 | CAR (AIH, IVF) |
|  |  | Free thyroxin (FT4) | 5.65 | CAR (AIH, IVF) |
|  |  | Cell blood count | 0.80 | Physiological delivery, preterm delivery, SC |
|  |  | CRP latex agglutination test | 3.17 | Physiological delivery, preterm delivery, SC |
| Other examination | | Acquisition of an oocyte and laparoscopic insertion of the oocytes and sperm in the fallopian tubes | 194.27 | CAR (AIH) |
|  |  | Artificial insemination – intracavital or intratubular | 45.19 | CAR (AIH) |
|  |  | Puncture of ovarian cysts –vaginal approach | 19.99 | CAR (AIH) |
|  |  | Ultrasound examination of pelvis | 15.05 | CAR (AIH, IVF) |
|  |  | IVF cycle with embryo transfer | 1,295.32 | CAR (IVF) |
|  |  | Cardiotocographic monitoring during delivery | 18.03 | Physiological delivery, preterm delivery, SC |
| Drugs | | Ovulation stimulators  (clomiphene / tamoxiphen) | 7.55 | CAR (AIH) |
|  |  | Follicle stimulating hormone | 275.17 | CAR (IVF) |
|  |  | General anesthesia (15 minutes) | 37.60 | SC, SpA |
| Days of hospitalization | | 1 bed-day in the dept of obstetrics and gynecology | 13.16 | Physiological delivery, preterm delivery, SC |
|  |  | 1 bed-day in an intensive care unit | 47.00 | SpA, SC |

The costs for medical check-ups, laboratory tests and other examinations were calculated by using payment algorithm (Decree No. 439/2008) and by using data extracted from the legislation administered by the Czech Ministry of Health (Decree No. 411/2011; Decree No. 439/2008). The costs of drugs administered were calculated by using the database of The State Institute of Drug Control in the Czech Republic (<http://www.sukl.cz/modules/medication/search.php?lang=1>). The costs of bed-days are the current costs applied in the in General University Hospital in Prague. SpA, spontaneous abortion; SC, cesarean section; CAR, Center of Assisted Reproduction; AIH, artificial insemination by husband; IVF, in vitro fertilization; CRP, C-reactive protein.
